# Proceedings of Southern and American Dental Conventions

**Published:** 1878-07

**Authors:** 


### BIBLIOGRAPHICAL.

---

*Proceedings of the American Dental Convention, the Southern Dental Association and the Dental Association of the State of Maryland and District of Columbia, in United Meeting,* held in Oakland, Md., August 14th to 17th, 1877.

The pamphlet of over 100 pages containing the proceedings as above, has just been received. It contains in addition to the article by Dr. Waterman, on the Spectroscope and Anæsthesia, published in the March No. of this JOURNAL, an address of welcome by Dr. Edward Nelson, of Frederick, Md.; a paper by Dr. E. Parsons, of Ga., on Dynamic or Vital Forces; one on Embryology, by Dr. Atkinson, of N. Y.; one on Therapeutical Treatment of Dentinal Pulps, by Dr. McLain, of New Orleans. On Anæsthesia, by Dr. H. C. Thompson, of Washington, D. C., besides various reports of committees, debates, etc. The report is neatly gotten up and contains much that is interesting to the profession.

Much debate seems omitted from the reported proceedings, whether by the official reporter, or by order of the association, does not appear,—much that appeared to the writer exceedingly interesting when heard.

The long delay in the appearance of this and of similar publications is apparently unnecessary, and must detract from the interest with which such papers are received. Ten months' delay in issuing a pamphlet of 100 pages is inexcusable. It would be better to make such abstracts from the proceedings as would give the gist of the debates, and furnish these and such such papers as are readable to the Journals.

The list of officers of the American Dental Convention is published as follows:

*President.*—Dr. J. Taft, of Cincinnati, Ohio.

*Vice President.*—Dr. J. R. Walker, of New Orleans, La.

*Secretary.*—Dr. Ambler Tees, of Philadelphia.

*Treasurer.*—Dr. J. G. Ambler, of New York.

A motion was adopted that a committee of ten be appointed to confer with the committee of ten of the American Dental Association, and a similar committee, if appointed, from the Southern Dental Association, with a view to the formation of a National Representative Dental Association, and the President, Dr. Atkinson, appointed on that committee Dr. R. B. Winder, of Baltimore; A. L. Northrop and John Allen, of New-York; B. F. Coy. of Baltimore; J. Taft, of Cincinnati; C. E. Kells, of New Orleans; R. B. Donaldson and H. B. Noble, of Washington; C. King, of Pittsburg; and C. F. Vandenburg, of ———

The officers of the Maryland and District of Columbia Dental Association are as follows:

*President.*—Dr. B. F. Coy, Baltimore.

*Vice President.*—Dr. J. C. Smithe, Washington.

*Recording Secretary.*—Dr. M. W. Foster, Baltimore.

*Reporting Secretary.*—Dr. F. F. Drew, Baltimore.

*Treasurer.*—Dr. H. B. Noble, Washington.

This Association meets in Washington, D. C., on the second Tuesdap, in October next.

